# Comparative evaluation of step-wise caries excavation and indirect pulp capping for vitality preservation in young permanent teeth: An *in vivo* Study

**DOI:** 10.6026/973206300213893

**Published:** 2025-10-31

**Authors:** Neetika Singh, Enna Singla, Charan Kamal Kaur, Salman Saleem, Reva Sharma, Ridhi Aggarwal

**Affiliations:** 1 Department of Pedodontics and Preventive Dentistry, Bhojia Dental College and Hospital, Baddi, Himachal Pradesh, India; 2 Department of Dentistry, Government Multispecialty Hospital, Chandigarh, India; 3 Department of Pedodontics and Preventive Dentistry, Gian Sagar Dental College and Hospital, Rajpura, Patiala, India

**Keywords:** Young permanent teeth, step-wise excavation, pulp capping

## Abstract

Management of deep carious lesions in young permanent teeth poses a significant clinical challenge due to the risk of pulpal exposure and subsequent loss of vitality. Therefore, this study evaluates and compares the clinical and radiographic outcomes of Step-wise Caries Excavation (SWE) versus Indirect Pulp Capping (IPC) in young permanent molars with deep carious lesions. Hence, a randomized controlled trial was conducted with 40 children aged 8-13 years, having 40 permanent molars with deep carious lesions, randomly allocated to either SWE (n=20) or IPC (n=20) groups. Clinical and radiographic evaluations were performed at baseline, 1 week, 3 months and 6 months. At 1-week follow-up, both groups showed similar success rates (95% in SWE versus 95% in IPC, p=1.000). Thus, we show that IPC is more effective than SWE in preserving pulp vitality in young permanent teeth with deep carious lesions, offering a less invasive and more economical treatment approach.

## Background:

Dental caries, as a chronic disease, is extremely common in people across the world and it is quite a problem in the young as well as the aged [1]. In young children or adolescents, the progression of caries into the young permanent teeth is more of a problem because their roots have not fully formed and it has thin dentinal walls [2]. The young permanent tooth has a proliferating, vascularized and cellular pulp, which is critical for the physis and its subsequent restorations [3]. Without the pulp, the roots are sure to underdevelop, which in turn, poses a risk to the overall structure of the teeth. Its prognosis is not good [4]. The management of deep carious lesions in young permanent teeth aims to preserve the pulp vitality while eliminating the carious tissue [5]. Conventional methods of caries removal usually result in the exposure of the pulp, thus leading to more complicated endodontic treatments [6]. This problem has led to the development of more conservative techniques, such as Step-wise Caries Excavation (SWE) and Indirect Pulp Capping (IPC) [7]. SWE takes a two-phase approach, at first removing only the top layer of carious dentin while leaving the deeper layer bordering the pulp. After duration of between 8 and 12 weeks, the cavity is accessed again and the remaining carious tissue is eliminated before the final restoration [8]. This is to ensure that the risk of pulp exposure during the first phase is minimized and that the pulp is allowed to form tertiary dentin [9]. Recent studies have shown conflicting results regarding the efficacy of these techniques. Many studies reported success rate with SWE over a 5-year follow-up period [10, 11-12]. In contrast, Maltz *et al.* (2012) found a 91% success rate with selective caries removal (similar to IPC) compared to 69% with stepwise excavation at 3 years [13]. A systematic review by Schwendicke *et al.* (2013) suggested that selective removal of soft dentin might be preferable to stepwise removal due to reduced risk of pulp exposure and fewer treatment sessions [14]. Despite these studies, there is still a lack of research on how effective SWE and IPC are specifically in young permanent teeth with deep carious lesions. Most previous studies have looked at either adult populations or primary teeth, with little research on children [15]. Additionally, the best duration for monitoring treatment success in young permanent teeth is not clearly defined [16]. Therefore, it is of interest to evaluate and compare the clinical and radiographic outcomes of SWE versus IPC in preserving the vitality of young permanent teeth with deep carious lesions in children aged 8-13 years over 6 months.

## Materials and Methods:

## Study design:

A randomized controlled trial was conducted to compare the efficacy of Step-wise Caries Excavation (SWE) and Indirect Pulp Capping (IPC) in preserving pulp vitality in young permanent teeth with deep carious lesions. The study followed the CONSORT guidelines for reporting clinical trials.

## Sample size calculation:

The sample size was calculated using G Power software version 3.1. Considering a power of 0.95, a significance level of 0.05 and expected success rates of 75% for IPC and 25% for SWE based on previous studies, the required sample size was determined to be 38 participants. This was rounded to 40 participants (20 in each group) to account for potential dropouts.

## Study population and setting:

The study was conducted in the Department of Pediatric and Preventive Dentistry at Bhojia Dental College and Hospital, Bhud, Baddi, Himachal Pradesh, India. Participants were recruited from the outpatient department between January 2022 and December 2022.

## Inclusion criteria:

Children aged 8-13 years with permanent first or second molars exhibiting deep carious lesions (Class I or Class II) were included. The teeth had to show signs of reversible pulpitis with a well-defined radio-opacity between the pulp and the carious lesion on radiographic examination. A positive response to pulp vitality testing was required for inclusion.

## Exclusion criteria:

Teeth with irreversible pulpitis, non-restorable teeth, periapical radiolucency, furcation involvement, widened periodontal ligament space, radiographic signs of internal or external resorption, or pulpal calcification were excluded. Children with special health care needs were also excluded from the study.

## Randomization and group allocation:

After screening 414 patients, 40 eligible participants were selected. Simple randomization was performed using 40 chits randomly allocated to Group I (SWE) or Group II (IPC), with 20 participants in each group. All treatment alternatives were discussed with parents/guardians and written informed consent was obtained before intervention.

## Ethical considerations:

The study was approved by the Institutional Ethical Committee of Bhojia Dental College and Hospital, Baddi, Himachal Pradesh (Approval No. BDC/BUDH/2903). The study was conducted in accordance with the Declaration of Helsinki.

## Clinical procedure:

All participants underwent complete scaling and polishing before the procedure. Topical anesthesia (15% lidocaine spray) was applied, followed by local anesthesia (2% lignocaine with 1:100,000 adrenaline) using a 27-gauge needle. Rubber dam isolation was achieved in all cases.

## Group I (Step-wise Excavation, n=20):

Superficial caries was removed using a slow-speed round diamond bur. Complete caries removal was performed from the walls using a spoon excavator, leaving soft carious dentin on the pulpal floor untouched. Calcium hydroxide (Dycal, Septodont) was applied over the carious dentin, followed by an interim restoration with glass ionomer cement (Ketac™ Molar). Vaseline was applied to prevent moisture contamination. Postoperative radiographs were taken. At the 3-month follow-up, after clinical and radiographic evaluation, the rubber dam was reapplied and the restoration was completely removed. The cavity was re-entered and any remaining soft caries was removed using a spoon excavator. Calcium hydroxide was reapplied, followed byglass ionomer cement. The cavity was then etched with 35% phosphoric acid (ThixoEtch) for 30 seconds, rinsed for 20 seconds and dried. A bonding agent (Ivoclar Vivadent) was applied and light-cured for 30 seconds. Permanent restoration was performed using composite resin (Ivoclar Vivadent) with an incremental technique. Occlusion was checked and adjusted as needed.

## Group II (Indirect Pulp Capping, n=20):

Infected and affected dentin was removed using a slow-speed round diamond bur, leaving only a thin layer of affected dentin on the pulpal floor. This layer was sealed with calcium hydroxide (Dycal, Septodont), followed by an interim restoration with glass ionomer cement (Ketac™ Molar). Vaseline was applied to prevent moisture contamination. Postoperative radiographs were taken. At the 3-month follow-up, after clinical and radiographic evaluation, the rubber dam was reapplied and the glass ionomer restoration was reduced to the dentinoenamel junction (2-3mm). The cavity was then etched with 35% phosphoric acid (ThixoEtch) for 30 seconds, rinsed for 20 seconds and dried. A bonding agent (Ivoclar Vivadent) was applied and light-cured for 30 seconds. Permanent restoration was performed using composite resin (Ivoclar Vivadent) with an incremental technique. Occlusion was checked and adjusted as needed.

## Follow-up schedule:

Clinical evaluations were performed at 1 week, 3 months and 6 months postoperatively. Radiographic examinations were conducted at baseline, 3 months and 6 months. Pulp vitality was assessed using an electronic pulp tester (Digitest) at each follow-up visit.

## Evaluation criteria:

Treatment success was determined based on clinical and radiographic criteria:

## Clinical Success:

[1] No tenderness to percussion or palpation

[2] Normal mobility

[3] No sinus tract formation

[4] Normal tooth function

[5] No signs of infection or swelling

[6] No evidence of subjective discomfort

[7] No signs of recurrent dental caries

## Radiographic Success:

[1] Formation of tertiary dentin

[2] Normal to slightly thickened periodontal ligament space (< 1 mm)

[3] Normal lamina dura in relation to adjacent teeth

[4] No evidence of resorption

[5] Absence of periapical radiolucency

Failure was defined as the presence of any of the following: persistent subjective symptoms, recurrent sinus tract or swelling, predictable discomfort to percussion or palpation, excessive mobility, progressive periodontal breakdown, progression of caries lesion to pulp, increased width of periodontal ligament space, intermittent and irregular lamina dura, evidence of resorption, or periapical radiolucency.

## Statistical analysis:

The statistical analysis was performed using SPSS software (IBM SPSS Statistics for Windows, Version 19.0, Armonk, NY, USA: IBM Corp.). Descriptive statistics were calculated as frequency and percentage for categorical variables and mean ± standard deviation (SD) for continuous variables. The comparison of clinical outcomes between the SWE and IPC groups at various time periods was carried out using the chi-square test. The level of significance for the study was set at a p-value of less than 0.05.

## Results:

A total of 40 permanent molar teeth were included in the study, with 20 teeth randomly allocated to each group. One tooth from each group failed at the 1-week follow-up. At the 3-month follow-up, 8 teeth in the SWE group and 2 teeth in the IPC group showed failure due to pain and swelling. At the 6-month follow-up, there were no new cases of failure in either group, maintaining the same failure rates as at 3 months. The demographic characteristics of the study participants are presented in Table 1 (see PDF). In the SWE group, there was an equal distribution of males and females (10 each, 50%). In the IPC group, 14 participants (70%) were males and 6 (30%) were females. Regarding arch distribution, the SWE group had an equal number of teeth in both arches (10 maxillary and 10 mandibular, 50% each), while the IPC group had 8 teeth (40%) in the maxillary arch and 12 teeth (60%) in the mandibular arch. The mean age of participants was 10.95±1.46 years in the SWE group and 10.60±1.87 years in the IPC group. At the 1-week follow-up, both groups showed similar success rates (Table 2 - see PDF). In the SWE group, 19 teeth (95%) were successful, while 1 tooth (5%) failed due to pain. Similarly, in the IPC group, 19 teeth (95%) were successful and 1 tooth (5%) failed due to pain. The difference between groups was not statistically significant (p=1.000). At the 3-month follow-up, a significant difference was observed between the groups (Table 2 - see PDF). In the SWE group, 12 teeth (60%) were successful, while 8 teeth (40%) failed due to pain and swelling. In the IPC group, 18 teeth (90%) were successful and 2 teeth (10%) failed due to pain and swelling. The difference between groups was statistically significant (χ^2^=4.800, p=0.028). At the 6-month follow-up, the results were consistent with the 3-month findings (Table 2see PDF). In the SWE group, 12 teeth (60%) remained successful, while 8 teeth (40%) continued to show failure. In the IPC group, 18 teeth (90%) were successful and 2 teeth (10%) showed failure. The difference between groups remained statistically significant (χ^2^=4.800, p=0.028). Radiographic evaluation at 6 months revealed tertiary dentin formation in 13 successful cases (65%) in the SWE group and 17 successful cases (85%) in the IPC group (Table 3 - see PDF). None of the failed cases in either group showed tertiary dentin formation. The difference in tertiary dentin formation between successful cases in the two groups was statistically significant (p=0.039). No cases of periapical radiolucency, resorption, or significant widening of the periodontal ligament space were observed in successful cases in either group at the 6-month follow-up.

## Discussion:

This study compared the efficacy of Step-wise Caries Excavation (SWE) and Indirect Pulp Capping (IPC) in preserving pulp vitality in young permanent teeth with deep carious lesions. The results demonstrated that while both techniques showed similar success rates at the 1-week follow-up (95% each), the IPC group had significantly better outcomes at 3 and 6 months (90% success) compared to the SWE group (60% success). These findings suggest that IPC may be a more effective approach for managing deep carious lesions in young permanent teeth. The higher success rate observed with IPC in our study aligns with previous research by Maltz *et al.* (2012), who reported a 91% success rate with selective caries removal (similar to IPC) compared to 69% with stepwise excavation at 3 years [13]. Similarly, a systematic review by Schwendicke *et al.* (2016) concluded that selective removal of soft dentin might be preferable to stepwise removal due to reduced risk of pulp exposure and fewer treatment sessions [14]. The lower success rate in the SWE group (60%) in our study could be attributed to several factors. First, the re-entry procedure at 3 months may have caused mechanical irritation to the pulp, leading to inflammation in some cases [17]. Second, the temporary restoration with glass ionomer cement may have allowed microleakage, permitting bacterial infiltration that compromised pulp vitality [18]. This is supported by our observation that most failures occurred after the interim restoration phase but before the permanent restoration was placed. The significantly higher rate of tertiary dentin formation in successful cases of the IPC group (85%) compared to the SWE group (65%) is noteworthy. Tertiary dentin formation is a key indicator of pulp health and reparative capacity [19]. The better performance of IPC in promoting tertiary dentin formation may be due to the more stable environment created by the single-stage procedure, allowing uninterrupted pulp healing and dentinogenesis [20]. In contrast, the two-stage SWE approach might have disrupted this process during the re-entry phase. Our findings contrast with those of Bjørndal *et al.* (2017), who reported a 60.2% success rate with SWE over a 5-year follow-up period [12]. The discrepancy could be explained by differences in study populations, follow-up duration and success criteria. Coll *et al.* studied adult patients, whereas our research focused on children and adolescents with developing permanent teeth, which may have different healing responses [21]. The gender distribution in our study showed a higher proportion of males in the IPC group (70%) compared to the SWE group (50%). However, this difference was not statistically significant and unlikely to have influenced the outcomes, as previous studies have not shown gender-based differences in pulp healing responses [22]. The arch distribution revealed a higher proportion of mandibular teeth in the IPC group (60%) compared to the SWE group (50%). Mandibular molars generally present greater challenges in moisture control during restorative procedures, which could potentially affect treatment outcomes [23]. Despite this, the IPC group still showed better results, further supporting the efficacy of this technique. Between groups, the mean age of participants was similar (10.95±1.46 years in SWE vs. 10.60±1.87 years in IPC). The range of age represents a vital period for root development in permanent molars [24]. Vitality of pulp during this stage needs to be preserved for normal root maturation and apical closure [25]. A comparable randomized clinical trial by Manhas *et al.* (2020) also demonstrated that Indirect Pulp Capping produced higher clinical success and better pulpal response than Step-wise Caries Excavation in permanent teeth of pediatric patients, reinforcing the superiority of IPC as a minimally invasive approach [26]. The present study suggested IPC may be particularly beneficial in this age group, as it minimizes disruption to the pulp and allows continued root development. IPC offers several advantages over SWE: it requires only one treatment session, eliminates the need for re-entry, reduces patient discomfort and is more economical [27]. These factors are particularly vital, where patient compliance and treatment acceptance can be challenging [28]. However, the present study has some shortcomings. The follow-up period was relatively short (6 months) and longer-term studies are needed to assess the durability of the results. In addition, thickness measurement of the remaining dentin beneath the carious lesions may also influence treatment outcomes [29].

## Conclusion:

Indirect Pulp Capping (IPC) is more effective than Step-wise Caries Excavation (SWE) in preserving pulp vitality in young permanent teeth with deep carious lesions is shown. IPC offers several advantages over SWE, including requiring only one treatment session, eliminating the need for re-entry, reducing patient discomfort and being more economical. Thus, we show that IPC should be considered as the treatment of choice for managing deep carious lesions in young permanent teeth, particularly in the pediatric population, where patient cooperation and treatment acceptance are important considerations.
